# Identification of immune characteristic biomarkers and therapeutic targets in cuproptosis for sepsis by integrated bioinformatics analysis and single-cell RNA sequencing analysis

**DOI:** 10.1016/j.heliyon.2024.e27379

**Published:** 2024-03-03

**Authors:** Tianfeng Wang, Xiaowei Fang, Ximei Sheng, Meng Li, Yulin Mei, Qing Mei, Aijun Pan

**Affiliations:** a Department of Critical Care Medicine, The First Affiliated Hospital of USTC, Division of Life Science and Medicine, University of Science and Technology of China, Hefei, Anhui Province, 230001, China; b WanNan Medical College, Wuhu, Anhui, 241002, China; c Department of Intensive Care Unit, The Affiliated Provincial Hospital of Anhui Medical University, Anhui, 230001, China

**Keywords:** Sepsis, Cuproptosis, Immune infiltration, Machine learning, ceRNA network, scRNA sequencing

## Abstract

**Background:**

Cuproptosis is a copper-dependent cell death that is connected to the development and immune response of multiple diseases. However, the function of cuproptosis in the immune characteristics of sepsis remains unclear.

**Method:**

We obtained two sepsis datasets (GSE9960 and GSE134347) from the GEO database and classified the raw data with R packages. Cuproptosis-related genes were manually curated, and differentially expressed cuproptosis-related genes (DECuGs) were identified. Afterwards, we applied enrichment analysis and identified key DECuGs by performing machine learning techniques. Then, the immune cell infiltrations and correlation between DECuGs and immunocyte features were created by the CIBERSORT algorithm. Subsequently, unsupervised hierarchical clustering analysis was performed based on key DECuGs. We then constructed a ceRNA network based on key DECuGs by using multi-step computational strategies and predicted potential drugs in the DrugBank database. Finally, the role of these key genes in immune cells was validated at the single-cell RNA level between septic patients and healthy controls.

**Results:**

Overall, 16 DECuGs were obtained, and most of them had lower expression levels in sepsis samples. Afterwards, we obtained six key DECuGs by performing machine learning. Then, the LIPT1-T-cell CD4 memory resting was the most positively correlated DECuG–immunocyte pair. Subsequently, two different subclusters were identified by six DECuGs. Bioinformatics analysis revealed that there were different immune characteristics between the two subclusters. Moreover, we identified the key lncRNA OIP5-AS1 within the ceRNA network and obtained 4 drugs that may represent novel drugs for sepsis. Finally, these key DECuGs were statistically significantly dysregulated in another validation set and showed a major distribution in monocytes, T cells, B cells, NK cells and platelets at the single-cell RNA level.

**Conclusion:**

These findings suggest that cuproptosis might promote the progression of sepsis by affecting the immune system and metabolic dysfunction, which provides a new direction for understanding potential pathogenic processes and therapeutic targets in sepsis.

## 1 Introduction

Sepsis is defined as a systemic excessive immune-inflammatory response caused by dysfunctional reactions to infection, which can lead to multiple organ dysfunction syndrome (MODS), which seriously threatens the life safety of patients and has a high mortality rate [1,2]. The pathogenesis of sepsis is extremely complex. In recent years, immune disorders, dysfunction of immune homeostasis, apoptosis of immune cells, excessive inflammatory reactions and abnormal regulation of molecules have attracted much attention and are involved in the initiation mechanism of sepsis [3,4]. Anti-inflammatory drugs are increasingly available to treat sepsis, but their effectiveness remains limited, and few have been shown to be clinically effective in reducing patient outcomes [5,6]. Clinicians are faced with severe challenges in the diagnosis, treatment and management of sepsis patients [7]. Although early diagnosis of sepsis is very important for quick treatment and improvement of prognosis in sepsis patients [8], at present, there is a lack of early and effective diagnosis and intervention for sepsis [9,10]. Therefore, a comprehensive and in-depth analysis of the pathogenesis of sepsis and finding specific biomarkers related to sepsis will provide a new strategy for the early clinical diagnosis and treatment of sepsis, which could be important for reducing sepsis mortality and providing insight into the aetiology of sepsis.

In general, programmed cell death includes pyrosis, apoptosis, necrosis and NETosis [11]. Iron-dependent cell death was discovered in 2012 and then applied in the study of sepsis [12]. However, in addition to ferrum, other metal ions, such as copper and zinc, are also defined as basic trace elements that are related to many biological processes, such as mitochondrial respiration, autophagy, antioxidant processes and kinase signalling [13]. Recently, attention has been given to cuproptosis, which is a novel mode of cell death induced by copper [14]. Cuproptosis is a copper-dependent cell death caused by the direct binding of copper to the fatty acylation component of the tricarboxylic acid cycle in the mitochondrial respiratory chain, which causes the accumulation of fatty acylated proteins and subsequent loss of iron-sulfur cluster proteins, leading to eventual cell death [14,15]. Copper ion maintenance can be used as a cofactor of enzymes, and copper homeostasis is mainly regulated by mitochondria [16]. Mitochondrial functions and metabolism play crucial roles in immune regulation and inflammatory activation in various diseases [17]. Previous studies have shown an increase in serum Cu^2+^ concentrations in multiple tumour patients compared with normal controls [18,19]. Jiang et al. established the cuproptosis-related gene risk score prognostic model, and a higher cuproptosis score suggested worse survival, and the score was positively related to immune infiltrates in the tumour microenvironment of oesophageal carcinoma [20]. In addition, Nie et al. found that disulfiram is an aldehyde dehydrogenase inhibitor and could be used as a drug to treat cancers and infectious diseases via cuproptosis [21]. These studies suggest that cuproptosis is closely related to the development and pathogenesis of diseases. However, cuproptosis in the pathogenesis of sepsis is still unclear.

Here, to explore the immune characteristics and biological functions of cuproptosis-related gene regulation in sepsis, we first used the GEO database to identify the genes that were dysregulated between sepsis and healthy samples. We then obtained differentially expressed cuproptosis-related genes (DECuGs) by the intersection of the differentially expressed genes and cuproptosis-related genes. Multiple machine learning techniques were performed to identify the key DECuGs. Next, unsupervised hierarchical clustering analysis was performed by the expression levels of the six key DECuGs, and 210 sepsis samples were divided into the two distinct DECuGs patterns in sepsis. Furthermore, the differences in circulating immune cells between different subclusters were analysed, and the biological function of GSVA between the two patterns was employed. WGCNA was then performed to explore crucial modules associated with DECuG patterns. Then, we analysed lncRNA-mediated ceRNAs based on key DECuGs and identified potential therapeutic drugs that could be used to treat sepsis. We validated these key DECuGs in the GSE32707 validation cohort and generated ROC curves for the diagnostic and prediction effectiveness of candidate key DECuGs in the validation cohort. Finally, the role of these key genes in immune cell features was analysed by single-cell RNA sequencing. In conclusion, our study is the first to systematically analyse the roles of cuproptosis in sepsis, which could provide us with novel insight to better understand the molecular mechanism in the pathogenesis of sepsis and discover biomarkers for the diagnosis and treatment of sepsis.

## 2 Materials and methods

### 2.1 Datasets and cuproptosis-related gene acquisition

Three raw datasets of gene expression profiles for sepsis patients and controls (GSE9960, GSE134347 and GSE32707 from the GEO database (https://www.ncbi.nlm.nih.gov/geo/)) used in the present study were obtained by the “GEOquery” R package. The GSE9960 dataset includes 54 sepsis samples and 16 normal controls, the GSE134347 dataset includes 156 sepsis samples and 83 normal controls, and the GSE32707 dataset includes 41 sepsis samples and 34 normal controls. The GSE9960 and GSE134347 datasets were used to identify dysregulated genes by the “SVA” R package to eliminate batch effects and normalize for correction datasets. The GSE32707 dataset was used to validate the results. Data were transformed into log2 by using R software (version 4.1.3) for subsequent analyses. In addition, we obtained 63 genes that were involved in cuproptosis by combining genes relevant to cuproptosis from a prior literature [14], MsigDB (v7.0) [22] and FerrDb [23] databases.

### 2.2 Identification of dysregulated genes and cuproptosis-related genes in sepsis

The differentially expressed genes (DEGs) between sepsis and healthy controls in the combined dataset were identified by using the “limma” R package, and the thresholds of |Fold Change (FC)|>1.2 and P < 0.05 were chosen as statistically significant. Due to the specificity of datasets and large sample sizes, setting a lower FC threshold can reveal that even minor gene expression changes may be statistically significant and biologically relevant in these contexts. Therefore, we have set the FC threshold to 1.2. Furthermore, the P-values are adjusted P-values corrected using FDR method. The cuproptosis-related genes were mapped with DEGs, and the intersection genes were differentially expressed cuproptosis genes (DECuGs) and defined as sepsis-related DECuGs. The functional annotation (GO and KEGG pathway analysis) of DECuGs was performed by using the “clusterProfiler” R package [24] to further explore the biological functions of DECuGs. Furthermore, the “corrplot” and “circlize” R packages were used to estimate the correlation between DECuGs.

### 2.3 Machine learning for screening feature DECuGs

Based on the DECuGs acquired through mapping DEGs and cuproptosis-associated genes, we applied two machine learning techniques to filter the key feature DECuGs as biomarkers for sepsis diagnosis. Previous research has found that in the field of biomedical sciences, machine learning is highly efficient in identifying disease-related biomarkers, making accurate clinical diagnoses, and determining optimal treatment strategies [25]. The advent of machine learning can unlock large biomedical and patient datasets, which is conducive to new paradigms in the diagnosis and treatment of various diseases [26]. The least absolute shrinkage and selection operator (LASSO) algorithm [27] and support vector machine recursive feature elimination (SVM-RFE) [28] algorithm were carried out to identify the feature DECuGs between sepsis and healthy controls based on the “glmnet”, “e1071” and “kernlab” R packages. Compared to some other machine learning methods, LASSO and SVM demonstrate good adaptability and generalization ability across various datasets, particularly maintaining high performance on small-sample datasets. The final candidate key DECuGs were obtained by the intersection of two algorithms for sepsis diagnosis. The expression levels of candidate key DECuGs were further tested in the validation cohort. In addition, the “circlize” R package was used to estimate the correlation between key DECuGs. The “pROC” R package was used to analyse the receiver operating characteristic curve (ROC) for further detecting the prediction effectiveness of the model in distinguishing sepsis from nonsepsis controls.

### 2.4 Exploring the correlation between DECuGs and immune cell infiltration

We performed 22 kinds of circulating immune cell distribution based on the CIBERSORT algorithm (https://cibersortx.stanford.edu) via the provided R script by using 1000 permutations to estimate the relative abundance of immune cell infiltration. The functions of v-SVR were implemented by the “e1071” R package. We filtered the samples for P < 0.05 in CIBERSORT as statistically significant results. Next, we analysed the correlation between DECuGs and infiltrating immune cells by R software. Finally, these results were visualized based on the “reshape2” and “ggpubr” R packages.

### 2.5 Analysis of gene set enrichment (GSEA)

We performed GSEA of key DECuGs by the “c2. cp.kegg.symbols” file obtained from the MSigDB database to identify their biological significance and functions. After 100 permutations, a gene set was considered to be significantly enriched at a threshold of P < 0.05 and FDR value < 0.25.

### 2.6 Subcluster analysis of key DECuG expression patterns

Unsupervised hierarchical clustering analysis was performed on the 210 sepsis samples by the expression level of the six key DECuGs to estimate the cluster numbers and robustness based on using the “ConsensusClusterPlus” R package [29]. Then, a principal component analysis (PCA) plot was generated to determine the geometrical distance and expression status between different subclusters. Furthermore, the expression levels of DECuGs in the two subclusters were determined and are shown using boxplots and heatmaps (P < 0.05 was considered statistically significant). The differences in circulating immune cells between different expression subclusters were compared by R software.

### 2.7 Biological function and module analysis of different subclusters

Gene set variation analysis (GSVA) [30] was employed to illustrate the functional distinction between two subclusters by the “c2. cp.kegg.symbols” and “c5. go.symbols” files obtained from the MSigDB database. The bar plot was used to show that the two clusters of DECuGs differed from each other in terms of the pathway activation score (P < 0.05 considered statistically significant). Moreover, to identify the significant modules related to different subclusters, we applied weighted gene coexpression network analysis (WGCNA) via the “WGCNA” R package [31]. Based on WGCNA, all samples were clustered to check whether there were obvious outliers, and then the coexpression network was constructed based on paired Pearson correlation coefficient matrices using the WGCNA method. The minimum dynamic tree cutting algorithm was used to cluster genes into different functional modules. Module membership (MM) and gene significance (GS) were calculated to determine the modules associated with the different subclusters. Finally, the important module gene information was extracted for further analysis.

### 2.8 Hypergeometric test and coexpression correlation analysis for ceRNA interactions

Next, we constructed a ceRNA network with key DECuGs based on the ceRNA theory that competing mRNA–lncRNA interaction pairs share miRNA-binding sites [32]. Then, we identified mRNA‒miRNA interaction pairs using the miRTarBase (Release 8.0) database [33]; mRNA‒miRNA interactions were then filtered by key DECuGs for sepsis. LncRNA-miRNA interactions were extracted from starBase [34], DIANA-LncBase [35], and LncACTdb [36]. Then, we used hypergeometric tests to identify competing pairs based on the common miRNAs of any pair of mRNAs and lncRNAs; P-values were computed using the formula given in Equation [1]:

(1) $$P=1-\sum_{k=0}^{x}\frac{\left(\begin{array}{l} m\\ k \end{array}\right)\left(\begin{array}{l} N-m\\ n-k \end{array}\right)}{\left(\begin{array}{l} N\\ n \end{array}\right)}$$

For each interaction pair, N denotes the total number of miRNAs in the interaction data, n and m denote the number of miRNAs that were associated with one mRNA and one lncRNA, and x represents the number of miRNAs shared with the mRNA and lncRNA. mRNA‒lncRNA competitive interaction pairs with P-value <0.05 were considered to represent potential ceRNA pairs. Next, to identify lncRNA‒mRNA interaction pairs, we applied coexpression correlation analysis to the lncRNA-mRNAs by using Pearson correlation coefficients (PCC) to examine the expression patterns of genes and lncRNAs. The expression data of the genes and lncRNAs were downloaded from the Genotype-Tissue Expression (GTEx, v8 release) [37] and calculated by using the following formula given in Equation [2]:

(3) $$\rho_{X,Y}=\frac{cov\left(X,Y\right)}{\sigma_{X}\sigma_{Y}}$$

Finally, the coexpressed mRNA–lncRNA pairs that met the hypergeometric test threshold (P-value<0.01) and crossed the PCC threshold (PCC >0.3 and P-value<0.01) were regarded as statistically significant interaction pairs.

### 2.9 Key DECuG expression levels in validation GEO datasets

As described previously, GSE32707 downloaded from GEO was used to validate the key DECuGs. The “limma” R package was applied again to identify DEGs in GSE32707. Finally, we generated a receiver operating characteristic curve for the diagnostic effectiveness of candidate key DECuGs and prediction effectiveness of the model in distinguishing sepsis from nonsepsis controls in the GSE32707 validation cohort.

### 2.10 Single-cell RNA-seq (scRNA-seq) analysis

We obtained the scRNA-seq count matrix (GSE167363) from the GEO database. GSE167363, containing 5 sepsis patients and 2 healthy controls, was selected for analysis. Quality control was performed, and expression matrix files were generated based on gene and UMI counts by the R Seurat (version 3.1.2) package [38]. The cells were removed based on gene counts between 200 and 5000 and UMI counts below 30,000. Then, Harmony was used for batch correction. We selected the top 2000 variable genes, and cells were separated into the 14 most significant principal components by the Find Clusters function in the Seurat package. A 2D uniform manifold approximation and projection (UMAP) was adopted for display. Cluster annotations were analysed by comparing cells in different clusters to annotated reference datasets based on the SingleR package [39].

### 2.11 Statistical analysis

Data and statistical analysis were performed by R software (version 4.1.3). The Wilcoxon test was used to compare the significant differences between the two groups. Spearman correlation was used to evaluate the relationship between the expression levels of DECuGs and circulating immune cells. A P-value<0.05 was considered statistically significant.

## 3 Results

### 3.1 Integrating GEO data and identification of DEGs and the expression patterns of DECuGs

**Fig. 1:**
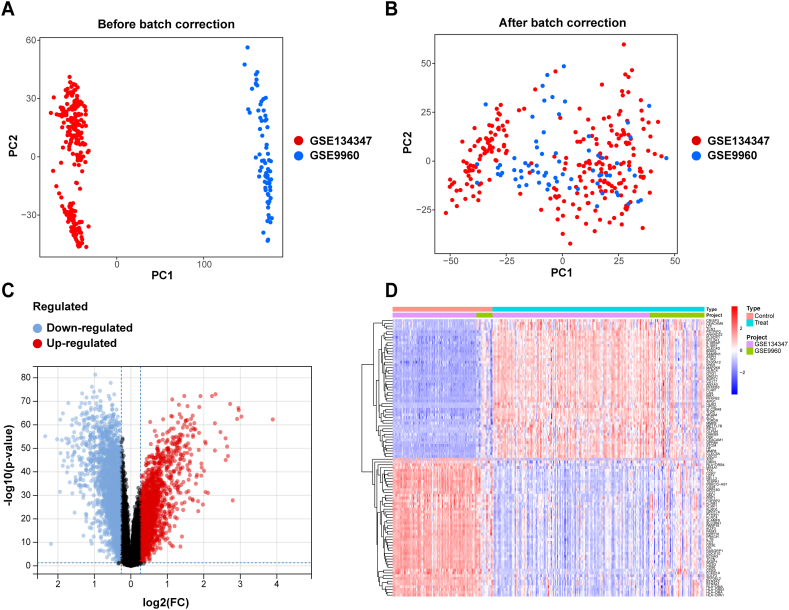
**GEO datasets integration and differentially expressed genes.** (A, B) PCA showed the batch effect between combined datasets before and after de-batching. (C) The volcano plot of DEGs in sepsis. (D) Clustered heatmap of the top 50 sepsis-related DEGs expression level. GEO, gene expression omnibus; PCA, principal component analysis; DEGs, differentially expressed genes.

**Fig. 2:**
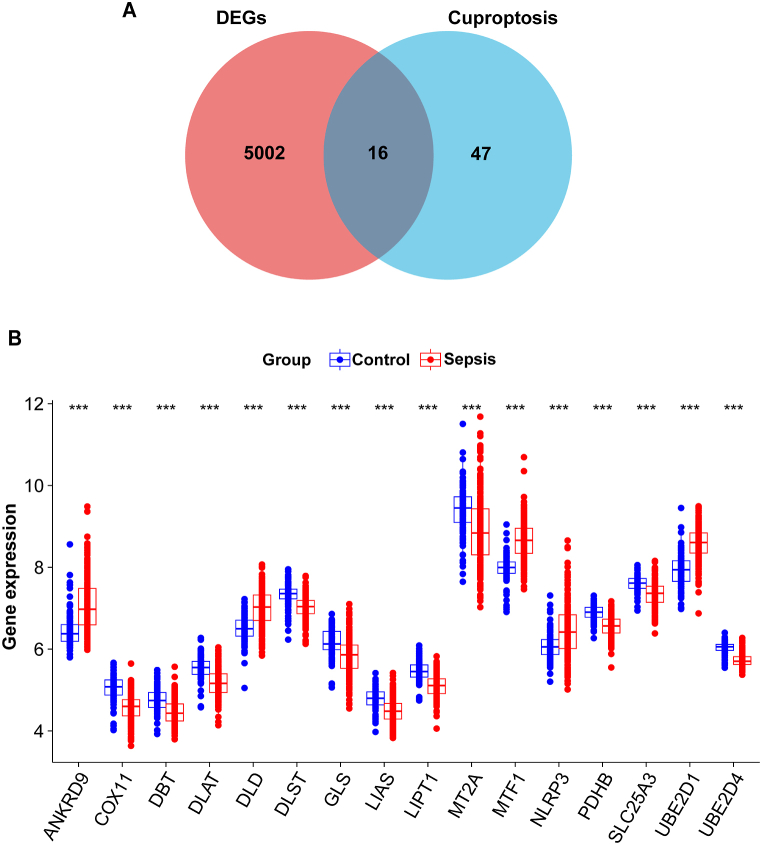
**Identification of DECuGs in sepsis.** (A) The intersection of genes between cuproptosis-related genes and DEGs. (B) The histogram of DECuGs overall expression in sepsis patients. *represents p < 0.05, **represents p < 0.01; ***represents p < 0.001; ****represents p < 0.0001.

The integrated expression profile from two GEO datasets (GSE9960 and GSE134347) that included 210 sepsis samples and 99 healthy controls. By preprocessing and removing the batch effects (Fig. 1A and B), we obtained a total of 5018 DEGs, including 1987 upregulated genes and 3031 downregulated genes, in sepsis samples by using the limma method. The marked differences in DEGs are presented in volcano plots and heatmaps (Fig. 1C and D). Then, the 16 DECuGs were obtained by the intersection of the 5018 DEGs and 63 cuproptosis-related genes (Fig. 2A). The expression patterns of DECuGs between sepsis samples and healthy samples are shown in Fig. 2B. Moreover, most of the DECuGs had lower expression levels.

### 3.2 Correlation and enrichment analysis of potential sepsis DECuGs

**Fig. 3:**
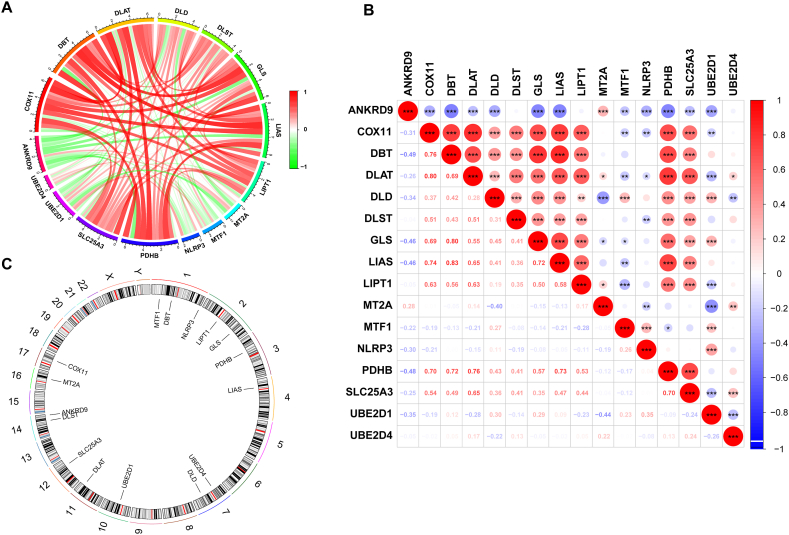
**Correlation analyses of potential DECuGs biomarkers in sepsis.** (A) The circos plot exhibiting the degree of correlation between the 16 DECuGs. (B) The heatmap exhibiting the correlation coefficients for 16 DECuGs, red and blue represent the positive correlations and negative correlations, respectively. (C) The relative positions of the 16 DECuGs on the chromosome. *represents p < 0.05, **represents p < 0.01; ***represents p < 0.001; ****represents p < 0.0001. (For interpretation of the references to colour in this figure legend, the reader is referred to the Web version of this article.)

**Fig. 4:**
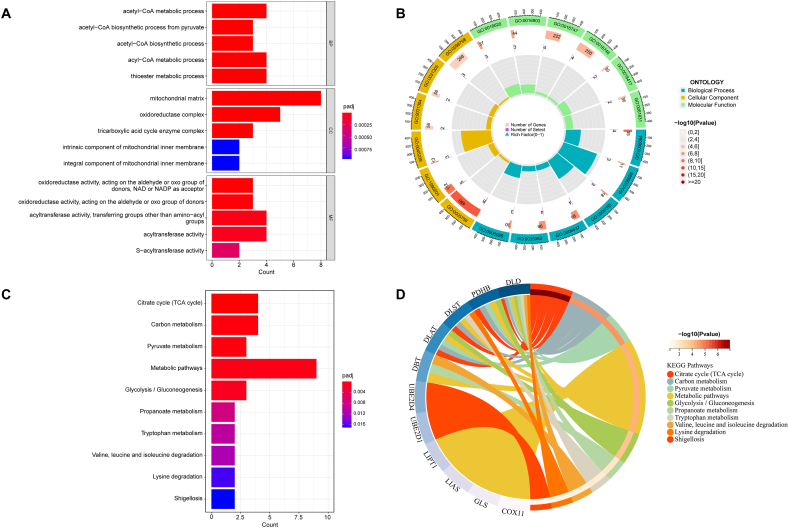
**Enriched items in GO and KEGG analysis by DECuGs.** (A) Bar plot of top 5 enriched items in GO (BP, MF and CC) analysis. (B) Circle plot of the top 6 items in GO (BP, MF and CC) analysis. (C) Bar plot of top 10 enriched items in KEGG pathway analysis. (D) Circle plot of correlation between KEGG pathways and involved genes.

After identifying the 16 DECuGs, a correlation analysis between these DECuGs was conducted by using Pearson's correlation coefficient (the threshold for the PCC is set at >0.3, with a P-value <0.05). The results showed that most DECuGs were highly correlated with each other (Fig. 3A and B). Furthermore, the chromosome positions of 16 DECuGs are visualized in Fig. 3C. We observed that DECuGs are distributed across chromosomes 1, 2, 3, 4, 7, 10, 11, 12, 14, 16, and 17. Notably, chromosome 1 has the highest number of genes, with three DECuGs located on it. Chromosome 1 is also known to contain many immune-related genes [40]. Previous research, including genome-wide association studies (GWAS) on sepsis patients, analysed genetic variations associated with 28-day survival rates, finding these variations across various chromosomal locations [41]. In present study, pinpointing each DECuGs location on the chromosomes provides new insights into the genetic basis of sepsis and may guide future clinical interventions. Then, we performed GO and KEGG enrichment analysis using the “clusterProfiler” R package to explore the potential functional roles of these DECuGs. The results of GO analysis revealed that these DECuGs were mainly in the enrichment of acetyl-CoA metabolic process and acetyl-CoA biosynthetic process from pyruvate related to the GO-BP terms (details in additional file 1: Table S1). In addition, in the cellular component category, enrichment of the mitochondrial matrix and oxidoreductase complex was discovered (Fig. 4A and B). The results of KEGG pathway (details in additional file 2: Table S2) analysis revealed that these DECuGs were mainly enriched in the citrate cycle (TCA cycle), metabolic pathways and glycolysis/gluconeogenesis (Fig. 4C and D).

### 3.3 Identification of the cuproptosis-related diagnostic genes by machine learning

**Fig. 5:**
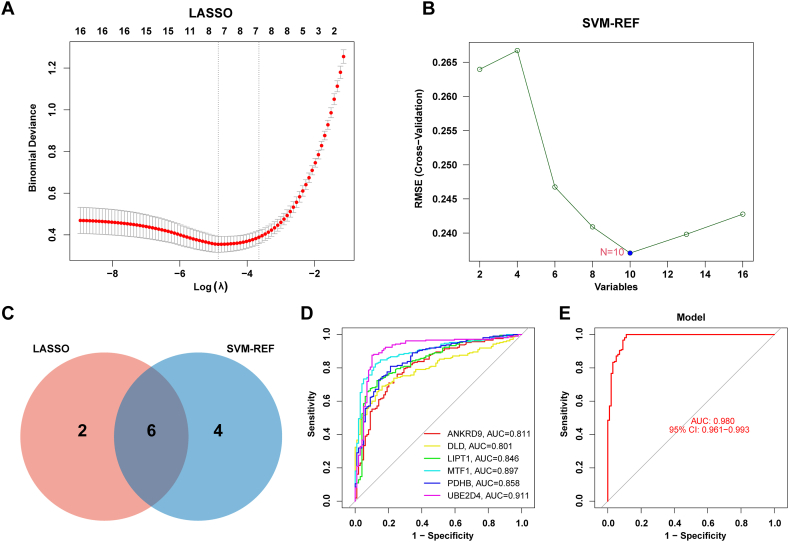
**Machine learning in the identification of key DECuGs.** (A, B) Identified optimal DECuGs by using LASSO regression and SVM algorithms. (C) The intersection of candidate DECuGs between LASSO and SVM algorithms. (D, E) ROC curves of biomarkers (six DECuGs and combined) in sepsis diagnosis.

After obtaining the 16 DECuGs, we established two different machine learning algorithms to identify potential diagnostic biomarkers of sepsis. As a result, 8 DECuGs as diagnostic biomarkers for sepsis were obtained by the LASSO regression algorithm (Fig. 5A). Meanwhile, 10 DECuGs were identified among the 16 DECuGs by the SVM-RFE algorithm (Fig. 5B). Finally, the 6 DECuGs (ANKRD9, DLD, LIPT1, MTF1, PDHB, UBE2D4) as diagnostic biomarkers for sepsis were identified by the intersection of the two machine learning algorithms (Fig. 5C). The ROC curves of 6 DECuGs were plotted to predict sepsis. Notably, UBE2D4 had the highest AUC (AUC = 0.911) among the 6 DECuGs (Fig. 5D). These results demonstrated that the 6 DECuGs signatures have excellent discrimination values (Fig. 5E).

### 3.4 Immune cell infiltration and correlation between key DECuGs and immune characteristics in sepsis

**Fig. 6:**
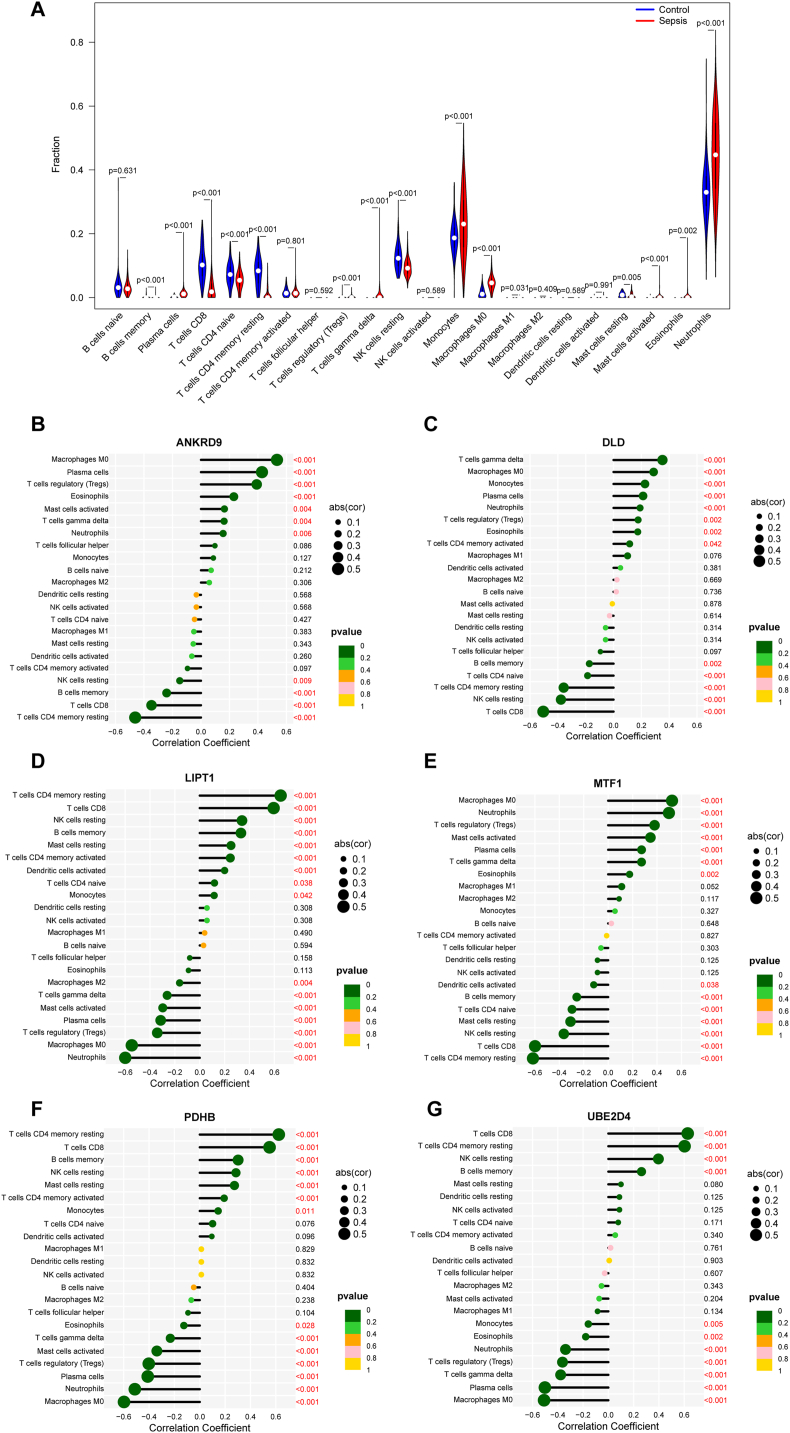
**Immune cell infiltration and correlation analysis between key DECuGs and immunocytes characteristics in sepsis.** (A) Violin plot of the differentially immune infiltrations in 22 types of immunocytes between sepsis patients and healthy controls. (B–G) Correlation analysis between key DECuGs (ANKRD9, DLD, LIPT1, MTF1, PDHB, UBE2D4) and immune infiltrating cells.

As we know that immune and inflammatory responses play important roles in the pathogenesis of sepsis, we performed immune cell infiltration to investigate the immunological regulation of sepsis. The violin plot showed that sepsis patients had a higher proportion of plasma cells, regulatory T cells (Tregs), gamma delta T cells, monocytes, M0 macrophages, M1 macrophages, activated mast cells, eosinophils and neutrophils and a lower proportion of memory B cells, CD8 T cells, naïve CD4 T cells, resting memory CD4 T cells, resting NK cells and resting mast cells (Fig. 6A). Then, as shown in Fig. 6B–G, the correlation between the six key DECuGs and 22 kinds of immunocytes was evaluated based on Spearman's correlation coefficient. The LIPT1-T cells CD4 memory resting was the most positively correlated DECuGs–immunocyte pair (r = 0.65, P-value<0.001), a higher expression level of LIPT1 and a higher infiltration of T cells CD4 memory resting cells in sepsis. The MTF1-T-cell CD4 memory resting cells were the most negatively correlated DECuG–immunocyte pair (r = −0.61, P-value<0.001), with a lower expression level of MTF1 and a lower infiltration of T-cell CD4 memory resting cells in sepsis.

### 3.5 GSEA of key DECuGs

**Fig. 7:**
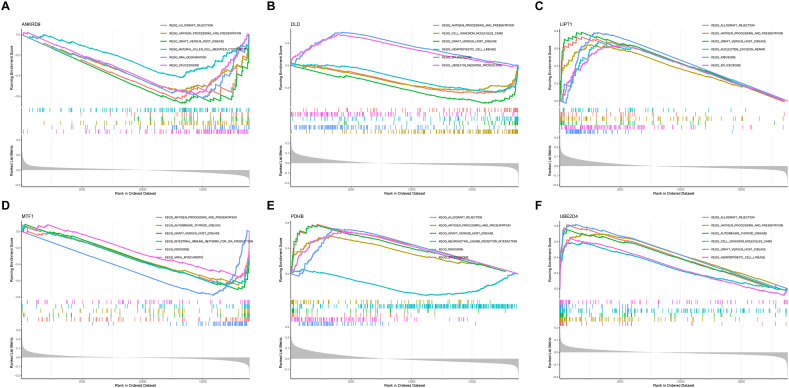
**The results of GSEA.** (A–F) GSEA investigation of ANKRD9, DLD, LIPT1, MTF1, PDHB and UBE2D4.

We conducted GSEA by R software to investigate the latent role of these key DECuGs, and the enriched results are shown in Fig. 7A–F. The GSEA results showed that most of these key DECuGs (ANKRD9, DLD, LIPT1, MTF1, PDHB and UBE2D4) were enriched in natural killer cell-mediated cytotoxicity, antigen processing and presentation, haematopoietic cell lineage and immune- and inflammatory-related pathways. These findings suggested that the six key DECuGs biomarkers could play important roles in the immune and inflammatory pathogenesis of sepsis.

### 3.6 Different clustering analysis of cuproptosis genes in sepsis

**Fig. 8:**
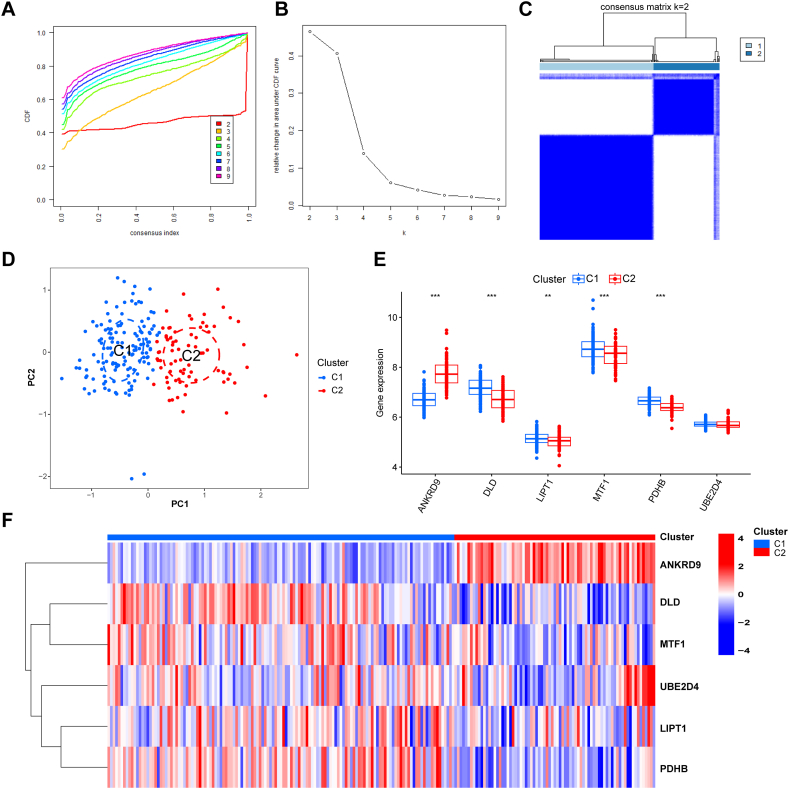
**Identification of the sepsis-subclusters based on key DECuGs.** (A) Representative cumulative distribution function (CDF) curve. (B) Representative CDF delta area curve. (C) Consensus clustering matrix when k is 2. (D) Visualization of the distribution of the two clusters by PCA analysis. (E) Boxplots showing differences in the expression of six key DECuGs between the two cuproptosis subclusters. (F) Heatmap showing the key DECuGs between the two cuproptosis subclusters.

Copper ion maintenance can be used as a cofactor of enzymes, and copper homeostasis is mainly regulated by mitochondria [16]. Mitochondrial functions and metabolism play crucial roles in immune regulation and inflammatory activation in patients with sepsis [42,43]. Thus, unsupervised consensus clustering was performed to divide the sepsis group into subclusters based on the six key DECuGs by using the “Consensus Cluster Plus” R package. We found that the consensus index of the CDF curve had the minimum fluctuation level and provided the most stable grouping when k = 2. As a result, we divided 210 sepsis samples into two distinct clusters, including 133 samples in Cluster 1 and 77 samples in Cluster 2 (Fig. 8A–C). In addition, the PCA of the two clusters showed that the 6 DECuGs could completely distinguish differences in transcriptome expression between the two clusters (Fig. 8D). Then, we compared different expression levels of 6 DECuGs in two clusters, and the boxplot and heatmap demonstrated the significance and distinction of gene expression patterns between the two clusters (Fig. 8E and F).

### 3.7 Immune characteristics between the two different cuproptosis expression clusters

**Fig. 9:**
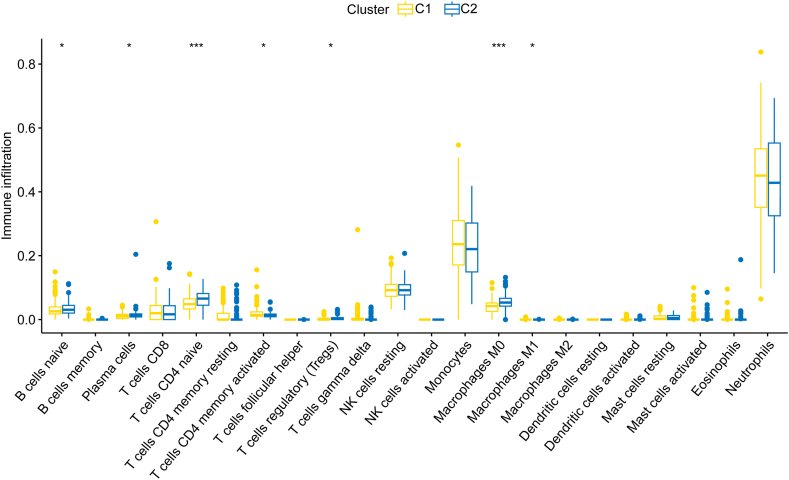
Boxplot of different immune features in two distinct cuproptosis subclusters.

To learn more about immune characteristics between the two different cuproptosis expression clusters, the infiltration of immunocytes was compared in the two clusters. We observed different immune infiltration characteristics between the two different clusters. We found that DLD, LIPT1, MTF1, PDHB and UBE2D4 were overexpressed in cluster-1, while ANKRD9 was overexpressed in cluster-2. The immune infiltration between the two clusters showed different statuses. Intriguingly, the proportions of CD4 memory activated T cells and M1 macrophages were greater in cluster-1, while the proportions of naïve B cells, plasma cells, naïve CD4 T cells, regulatory T cells and M0 macrophages were greater in cluster-2 (Fig. 9). The sepsis patients in cluster-2 were found to have higher infiltrating immunocytes, which indicated that cluster-2 possessed immune enrichment.

### 3.8 Biological functions between the two different cuproptosis expression clusters

**Fig. 10:**
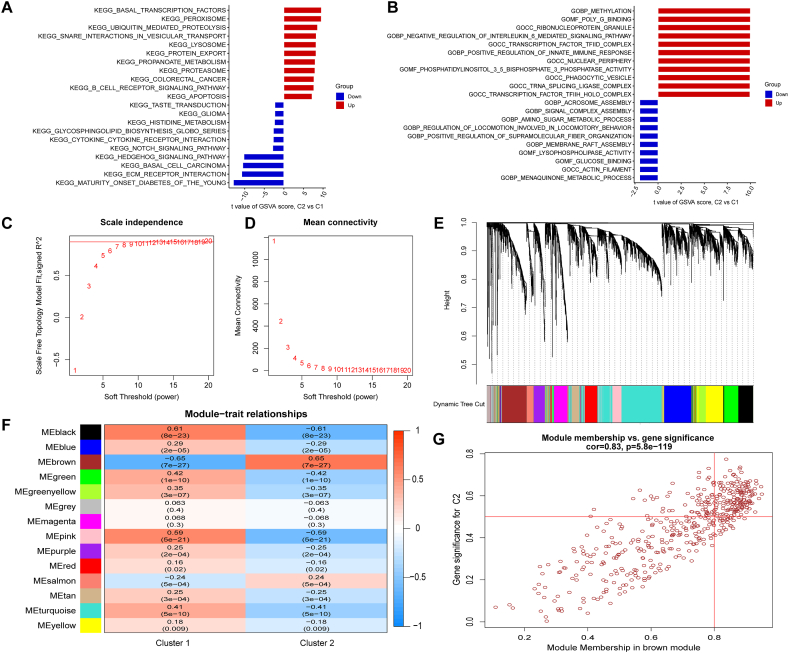
**Identification of biological function characteristics in two distinct cuproptosis subclusters.** (A, B) GSVA results of KEGG and GO gene sets in two distinct cuproptosis subclusters were plotted in bar plots. (C, D) Analysis of the scale free index and mean connectivity for different soft threshold powers. (E) Cluster dendrogram of the average linkage hierarchical clustering. (F) Heatmap displayed the correlation between different modules and the two cuproptosis subclusters. (G) The correlation scatterplot of gene significance for sucluster-2 versus module membership in the brown module. (For interpretation of the references to colour in this figure legend, the reader is referred to the Web version of this article.)

GSVA enrichment was analysed between two different pyroptosis expression clusters. Through GSVA, we found that the KEGG pathways involved in basal transcription factors, peroxisomes, ubiquitin-mediated proteolysis and B-cell receptor signalling pathways were higher in cluster-2, and the pathways involved in ecm receptor interaction, hedgehog signalling pathway, notch signalling pathway and cytokine receptor interaction were higher in cluster-1 (Fig. 10A). Meanwhile, the gene functions involved in methylation, negative regulation of the IL6-mediated signalling pathway, and positive regulation of the innate immune response were higher in cluster-2, and the signal complex assembly and positive regulation of supramolecular fibre organization were higher in cluster-1 (Fig. 10B). The GSVA function analysis in different clusters indicated that cluster-2 exhibited relatively higher enrichment levels of immune responses and immune-related pathways, with higher levels of immunocytes and lower expression levels of DECuGs. Next, we conducted a comprehensive gene landscape related to different cuproptosis expression clusters based on the WGCNA to identify modules correlated with different regulation of cuproptosis-related genes (Fig. 10C–E). Finally, 14 gene modules were obtained, and the correlation between the two different cuproptosis expression clusters and modules was analysed (Fig. 10F). Moreover, gene modules related to cuproptosis-related expression clusters were identified by using WGCNA, and the results showed that Cluster 2 was the most positively correlated with the brown module, and Cluster 1 was the most negatively correlated with the brown module (Fig. 10G).

### 3.9 Construction of the ceRNA network and prediction of potential drugs for the treatment of sepsis

**Fig. 11:**
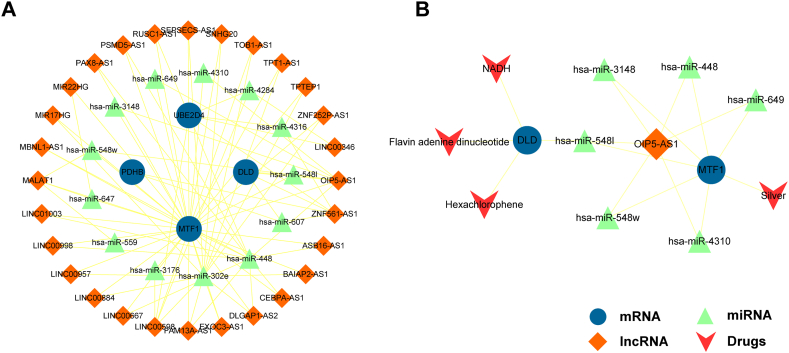
**Construction of ceRNA subnetwork and associated drugs.** (A) Construction of ceRNA network based on key DECuGs. (B) Identified potential drugs targeted OIP5-AS1 mediated ceRNAs. Blue circle represents mRNAs, green triangle represents miRNAs, orange diamond represents lncRNAs and red “V” represents drugs. (For interpretation of the references to colour in this figure legend, the reader is referred to the Web version of this article.)

We used key DECuGs to reconstruct a ceRNA network that contained 45 nodes, including 28 lncRNAs, 13 miRNAs and 4 mRNAs (Fig. 11A). For further analysis, all lncRNA nodes in the ceRNA subnetwork were ranked in descending order in terms of their degree and BC. Among them, the lncRNA OIP5-AS1 had the highest degree distribution and was identified as the hub lncRNA. These findings suggested that the hub lncRNA-mediated ceRNAs are important regulators during the immunological and inflammatory pathogenesis of sepsis. Next, we performed drug analysis using our hub lncRNA-mediated ceRNA subnetwork. We identified the coexpression of the lncRNA OIP5-AS1 with DECuGs from the ceRNA network to identify potential drugs in the DrugBank database. As a result, we screened 4 potential drugs, silver, hexachlorophene, flavin adenine dinucleotide, and NADH, that were shown to be target by OIP5-AS1-mediated ceRNA regulator pairs (Fig. 11B). Collectively, the ceRNA network can serve as a starting point for investigations of the immune processes involved in sepsis, and our analysis identified novel therapeutic targets and drugs for the future treatment of sepsis.

### 3.10 Six key DECuGs expression levels and diagnostic values in validation GEO datasets

**Fig. 12:**
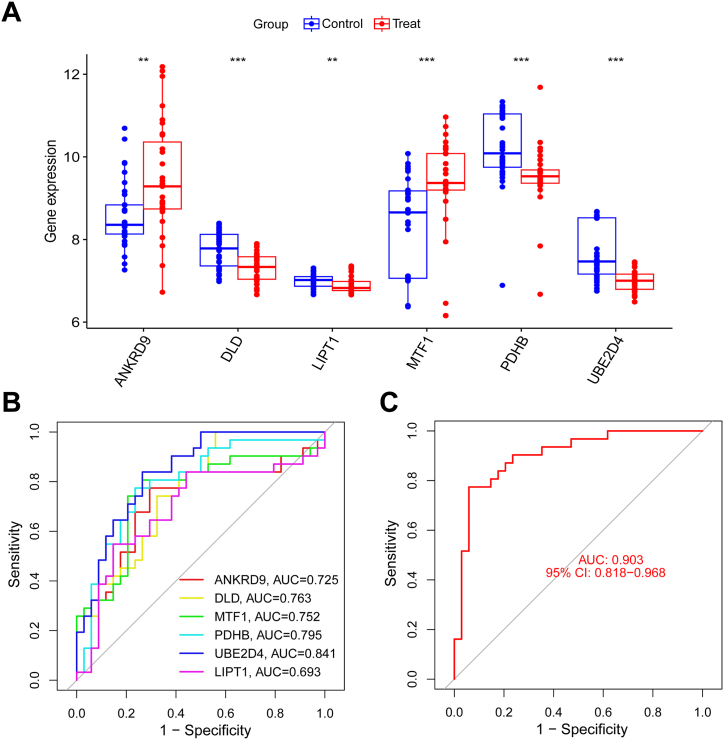
**Validation of the six key DECuGs in GSE32707 dataset.** (A) The histogram of DECuGs (ANKRD9, DLD, LIPT1, MTF1, PDHB, UBE2D4) expression between sepsis patients and normal controls in GSE32707. (B, C) ROC curves of biomarkers (6 DECuGs and combined) in the validation cohort. *represents p < 0.05, **represents p < 0.01; ***represents p < 0.001; ****represents p < 0.0001.

To further demonstrate the reliability of our results, the expression levels of six key DECuGs were validated in another gene expression profile (GSE32707) of sepsis. Next, by analysing the GSE32707 expression profile, 9550 DEGs were identified. As a result, the six key DECuGs were statistically significantly dysregulated in the validation set, and a boxplot of ANKRD9, DLD, LIPT1, MTF1, PDHB and UBE2D4 is shown in Fig. 12A (P-value<0.05). Moreover, the diagnostic values of these 6 DECuGs were determined in the GSE32707 validation cohort, and the ROC curves of the 6 DECuGs were plotted to predict sepsis (Fig. 12B). These results demonstrated that the 6 DECuG signatures had excellent discrimination values in the validation cohort (Fig. 12C). These findings further strengthen the credibility of our results.

### 3.11 Validation in a single-cell dataset

**Fig. 13:**
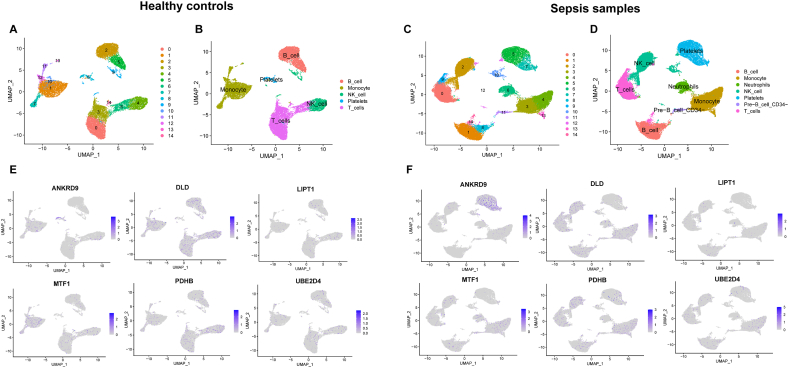
**Single-cell gene expression analysis of PBMCs in sepsis and normal control.** (A) and (C) UMAP plot of the cell clusters in healthy controls and sepsis samples. (B) and (D) UMAP plot exhibiting the cell clusters annotated by SingleR package in healthy controls and sepsis samples. (E) and (F) UMAP plot of the marker genes.

To further investigate the immune cell landscape of these key DECuGs at the single-cell level and cell subpopulations in sepsis, we obtained scRNA-seq data from the GSE167363 dataset, which contained peripheral blood mononuclear cells from 2 healthy controls and 5 patients with sepsis. As a result, we obtained 15912 cells conforming to quality control in healthy samples, and 20452 cells conformed to quality control in sepsis samples. Then, the cells were classified into 14 clusters (Fig. 13A–C). There were 7 main cell types identified from sepsis samples, including B cells, monocytes, neutrophils, NK cells, platelets, pre-B-cell CD34 and T cells (Fig. 13D). Moreover, six key DECuGs, including ANKRD9, DLD, LIPT1, MTF1, PDHB and UBE2D4, were analysed. These key DECuGs were later marked in cell types (Fig. 13F). The results of healthy controls are shown in Fig. 13B. DECuGs between healthy samples and sepsis samples in different cell types are shown in Fig. 13E. We found that the DECuGs within sepsis samples showed a major distribution in monocytes, T cells, B cells, NK cells and platelets. ANKRD9 was highly expressed in platelet cells. For immune cells, monocytes, T cells, B cells, and NK cells were related to DLD, LIPT1, MTF1, PDHB and UBE2D4 in the sepsis group. These key DECuGs in healthy samples showed a major distribution in T cells and monocytes.

## 4 Discussion

Sepsis is a systemic inflammatory response to infection that causes the dysregulation of immune system homeostasis [4]. For the past few years, with the improvement and development of diagnosis and treatment technology, the survival time of sepsis patients has been prolonged. However, the pathogenesis of sepsis is extremely complex and yet to be elucidated, and there is a lack of effective biomarkers for the timely diagnosis and treatment of sepsis patients. Therefore, we urgently need to explore the molecular mechanism of sepsis to further identify precision biomarkers and medications for sepsis. Recently, cuproptosis, a novel mode of cell death induced by copper, has attracted widespread attention in the research field. Previous studies have demonstrated the implications and therapeutic potential of targeting cuproptosis in various diseases [44]. To date, few studies have focused on cuproptosis in sepsis. Therefore, our study was the first to examine the landscape of cuproptosis in the sepsis field and identify potential therapeutic biomarkers through systematic bioinformatics analysis, which could provide a reliable direction for further experiments on sepsis in the future.

In the present study, we first used the GEO database to identify gene expression between sepsis patients and normal controls, and finally, we identified 16 DECuGs by the intersection of the 5018 DEGs and 63 cuproptosis-related genes. We used three raw datasets of gene expression profile for sepsis patients and controls (GSE9960, GSE134347 and GSE32707) from GEO database. The selection of these datasets is primarily based on several reasons. Firstly, both the GSE134347 and GSE9960 datasets consist of Peripheral Blood Mononuclear Cells (PBMCs) from patients. PBMCs include lymphocytes such as B cells and T cells, as well as monocytes, all of which play pivotal roles in the immune system. Sepsis is a systemic inflammatory response, and gaining a deep understanding of the behavior of these immune cells is crucial for studying the immune mechanisms underlying sepsis. Therefore, we focused on datasets that contain PBMCs as the sample type. In addition, the GSE134347 and GSE9960 datasets have a substantial number of samples: GSE134347 includes 156 sepsis patients and 83 healthy individuals, whereas the GSE9960 dataset comprises 54 sepsis patients and 16 healthy individuals. A larger sample size increases the reliability of the results and reduces the impact of random factors. GSE32707, used as a validation set, maintains consistency in sample type, containing 41 cases of sepsis and 34 healthy individuals. To further identify the roles and molecular mechanisms of cuproptosis-related genes in sepsis, we performed functional enrichment and correlation analyses. The major biological processes focused on acetyl-CoA biosynthetic process from pyruvate and mitochondrial matrix. According to previous studies, the pyruvate dehydrogenase complex plays key roles in the regulation of the remodelling of the tricarboxylic acid cycle, the release of inflammatory mediators and the balance of lactate in sepsis [45,46]. The most enriched KEGG pathway was metabolic pathways. A study confirmed that the metabolic pathways were related to immune cells and might play crucial roles in the pathogenesis of sepsis [47]. These findings partly explain the DECuGs involved in the pathogenesis of sepsis through regulating important biological processes and pathways. We discovered that the correlations among DECuGs provided strong evidence of antagonistic or synergistic effects in sepsis patients, which suggested that these DECuGs might participate in the development of sepsis together. Furthermore, we added a comparison of the correlation calculations between DECuGs and other genes, including non-DECuGs in DEGs or non-DECuGs in Cuproptosis-related genes (the threshold for the correlation coefficient is set at >0.3, with a P-value of <0.05). The results show that there is a higher proportion of significant correlations within the DECuGs group. The comparative analysis of correlations in the other two groups did not reveal higher correlations than those in the DECuGs group. Detailed information can be found in the additional file 3 and 4: Table S3 and Table S4.

Next, six key DECuGs (ANKRD9, DLD, LIPT1, MTF1, PDHB, UBE2D4) were identified as diagnostic biomarkers based on the two machine learning algorithms, suggesting their potential functional importance in the pathogenesis of sepsis. ANKRD9 is a gene involved in a variety of cellular processes, including the degradation of IMPDH2, and IMPDH2 is involved in the viral response pathway and considered a therapeutic target for COVID-19 treatment [48]. Zhou et al. found that the expression levels of DLD and LIPT1 in rheumatoid arthritis were higher than those in normal controls, and a diagnostic model was constructed based on genes that demonstrated their better predictive effect [49]. Another study showed that the MTF1 gene is an essential molecule for the mitochondrial Cu^+^ transporter PiC2, whose deficiency leads to dysfunction in the delivery of Cu^+^ to mitochondria and cytochrome *c* oxidase [50]. The PDHB gene could be a potential molecular biomarker for predicting tumour immune response [51]. Lee et al. identified that decreased expression of UBE2D family genes led to the accumulation of p53, which is an important pathway for renal toxic injury caused by cadmium [52]. However, the regulatory functions of these key DECuGs in sepsis have not been elucidated, and further studies are needed.

The immunocyte subpopulations might play a vital role in the pathogenesis of sepsis. Hence, we determined the correlations of six key DECuGs and immune subset infiltration between sepsis patients and healthy controls by the CIBERSORT algorithm. The relationship between six DECuGs and different immune cell abundance has been analysed. It has been turned out that these key DECuGs were closely linked to multiple immune cell infiltrations. According to the results, they might be crucial in predicting immunotherapy's effectiveness.

Based on the six key DECuGs, we then performed unsupervised hierarchical clustering analysis to identify different cuproptosis-related patterns. Immunosuppression and immune activation throughout the course of sepsis and dysfunction of the immune system could bring about persistent and unbalanced inflammatory and anti-inflammatory responses [53,54]. Furthermore, we analysed the key DECuGs for gene‐targeted drugs and the ceRNA network. We identified that the lncRNA OIP5-AS1-mediated ceRNA pair could represent a novel and highly promising diagnostic biomarker and therapeutic target for sepsis and four drugs that might be used to reduce inflammatory responses in sepsis treatment. Finally, the expression of these six DECuGs was validated in another validation cohort. All six diagnostic DECuG expression levels between sepsis samples and normal controls were proven. We constructed a classifier consisting of six key DECuGs, and the ROC curve demonstrated that the 6 DECuG signatures had excellent discrimination values in the validation cohort.

To further explore the cellular and molecular characteristics of each immune cell cluster involved in sepsis, we used the public scRNA-seq dataset to identify seven cell clusters in the PBMCs of patients with sepsis. The 6 DECuGs in each immune cell cluster were identified between healthy samples and sepsis samples, suggesting their important roles in inducing functional changes in different immune cells during sepsis. However, there were still some limitations in our methods. Firstly, the discrepancy in data types between the training and test sets presents a significant challenge in achieving high classification performance through independent testing. Secondly, our research represents a subsequent exploration and analysis of the GEO database, with metadata collected from various platform versions and some datasets lack clinical features. Due to the diverse sources and the considerable sample size, it is possible that batch effects might not have been completely eliminated. Finally, because of the small number of samples from patients with sepsis, some identified diagnostic risk genes may be false positives. It may be difficult to identify these diseases only from the differences in gene expression, which requires comprehensive analysis. In future studies, we will continue to concentrate and collecting experimental data to further explore the in vivo roles of these hub genes in sepsis pathogenesis. By using the alternative machine learning methods, it's possible to develop a classification model with improved performance, which could aid in the diagnosis of sepsis.

In summary, different cuproptosis-related gene clusters had distinct molecular and immune characteristics, which might help us improve the understanding of cuproptosis in sepsis patients. The classification method for clusters contributes to the revelation of disease heterogeneity and the molecular mechanism of sepsis, which could help us develop individualized therapies for sepsis. New drugs screened through the ceRNA network will provide novel insight into sepsis treatment. These results will encourage researchers to more deeply explore cuproptosis in sepsis and systematically reveal the pathogenesis of sepsis.

## 5 Conclusion

In conclusion, our work is the first to explore the biological functions and expression features of cuproptosis-related genes in sepsis and to identify the relationships between key DECuGs and immune cell infiltration. The heterogeneity and molecular mechanism of immunoinflammatory responses among sepsis patients with different cuproptosis patterns will provide us with novel insight into the underlying pathogenesis of sepsis. The model of six key DECuGs that were screened by the machine learning model could accurately classify sepsis patient subclusters and had excellent discrimination values in the diagnosis of sepsis patients. According to current research, the cuproptosis-related phenomenon exists in sepsis and is associated with immunocyte infiltration and metabolic pathway activity. The findings in our work did uncover the significant effects that cuproptosis-related genes have on the progression of sepsis and open a new direction for understanding the potential pathogenic processes and therapeutic targets in sepsis.

## Ethics approval and consent to participate

Not applicable.

## Consent for publication

Not applicable.

## Funding

This work was supported by the Scientific research project of Anhui Provincial Education Department (2022AH051264 and 2022AH051260) and the 10.13039/501100003995Natural Science Foundation of Anhui Province (2308085QH272).

## Data availability statement

The data used to analyse in this study could be found in the GEO database. The original contributions presented in the study are included in the article and supplementary material, further reasonable request can be directed to the corresponding author.

## CRediT authorship contribution statement

**Tianfeng Wang:** Writing – review & editing, Writing – original draft, Formal analysis, Data curation, Conceptualization. **Xiaowei Fang:** Methodology, Formal analysis, Data curation. **Ximei Sheng:** Visualization, Software. **Meng Li:** Visualization, Software. **Yulin Mei:** Validation, Software. **Qing Mei:** Writing – review & editing, Funding acquisition, Conceptualization. **Aijun Pan:** Writing – review & editing, Conceptualization.

## Declaration of competing interest

The authors declare they have no conflicts of interest.
