# Supplementary material for: Identification of immune characteristic biomarkers and therapeutic targets in cuproptosis for sepsis by integrated bioinformatics analysis and single-cell RNA sequencing analysis
Source: Heliyon. 2024 Mar 3;10(5):e27379. doi: 10.1016/j.heliyon.2024.e27379 (PMC10943398; doi:10.1016/j.heliyon.2024.e27379)
Supplement: Multimedia component 1 [file mmc1.docx]

**Supplementary files:**

**Supplementary file 1:**

Supplementary Table S1: The GO functional enrichment assessments of DECuGs.

| ONTOLOGY | ID | Description | pvalue |
| --- | --- | --- | --- |
| BP | GO:0006084 | acetyl-CoA metabolic process | 1.37609353009196e-08 |
| BP | GO:0006086 | acetyl-CoA biosynthetic process from pyruvate | 6.83784170027649e-08 |
| BP | GO:0006085 | acetyl-CoA biosynthetic process | 3.37025814204337e-07 |
| BP | GO:0006637 | acyl-CoA metabolic process | 8.48333766576937e-07 |
| BP | GO:0035383 | thioester metabolic process | 8.48333766576937e-07 |
| BP | GO:0006099 | tricarboxylic acid cycle | 1.66721784141126e-06 |
| BP | GO:0033865 | nucleoside bisphosphate metabolic process | 2.68350916476279e-06 |
| BP | GO:0033875 | ribonucleoside bisphosphate metabolic process | 2.68350916476279e-06 |
| BP | GO:0034032 | purine nucleoside bisphosphate metabolic process | 2.68350916476279e-06 |
| BP | GO:0044272 | sulfur compound biosynthetic process | 4.78201520082354e-06 |
| BP | GO:0006790 | sulfur compound metabolic process | 4.88755423746237e-06 |
| BP | GO:0035384 | thioester biosynthetic process | 5.78515370436162e-06 |
| BP | GO:0071616 | acyl-CoA biosynthetic process | 5.78515370436162e-06 |
| BP | GO:0009060 | aerobic respiration | 1.25857328646286e-05 |
| BP | GO:0033866 | nucleoside bisphosphate biosynthetic process | 1.38509070319554e-05 |
| BP | GO:0034030 | ribonucleoside bisphosphate biosynthetic process | 1.38509070319554e-05 |
| BP | GO:0034033 | purine nucleoside bisphosphate biosynthetic process | 1.38509070319554e-05 |
| BP | GO:0045333 | cellular respiration | 2.72278401309962e-05 |
| BP | GO:0006091 | generation of precursor metabolites and energy | 2.90705943729924e-05 |
| BP | GO:0043648 | dicarboxylic acid metabolic process | 5.68388615355322e-05 |
| BP | GO:0006878 | cellular copper ion homeostasis | 6.25269034302118e-05 |
| BP | GO:0006090 | pyruvate metabolic process | 7.63766522525518e-05 |
| BP | GO:0006103 | 2-oxoglutarate metabolic process | 9.09841852225964e-05 |
| BP | GO:0055070 | copper ion homeostasis | 9.09841852225964e-05 |
| BP | GO:0015980 | energy derivation by oxidation of organic compounds | 9.61353820649571e-05 |
| BP | GO:0071294 | cellular response to zinc ion | 0.000163673124813932 |
| BP | GO:0009150 | purine ribonucleotide metabolic process | 0.000168807481734472 |
| BP | GO:0009259 | ribonucleotide metabolic process | 0.000200749618821406 |
| BP | GO:0006163 | purine nucleotide metabolic process | 0.000223621806110966 |
| BP | GO:0019693 | ribose phosphate metabolic process | 0.000223621806110966 |
| BP | GO:0072521 | purine-containing compound metabolic process | 0.000269960735730805 |
| BP | GO:0009152 | purine ribonucleotide biosynthetic process | 0.000303507395340505 |
| BP | GO:0009260 | ribonucleotide biosynthetic process | 0.000377189485424135 |
| BP | GO:0046390 | ribose phosphate biosynthetic process | 0.000427795813186994 |
| BP | GO:0006164 | purine nucleotide biosynthetic process | 0.000434412996426781 |
| BP | GO:0006006 | glucose metabolic process | 0.000468489896857478 |
| BP | GO:0072522 | purine-containing compound biosynthetic process | 0.00049695729229962 |
| BP | GO:0009117 | nucleotide metabolic process | 0.000498921789500305 |
| BP | GO:0006753 | nucleoside phosphate metabolic process | 0.000530463706658739 |
| BP | GO:0019318 | hexose metabolic process | 0.000814245168731722 |
| BP | GO:0010043 | response to zinc ion | 0.000964958368994651 |
| BP | GO:0009165 | nucleotide biosynthetic process | 0.000994999601962382 |
| BP | GO:1901293 | nucleoside phosphate biosynthetic process | 0.00101780192215918 |
| BP | GO:0005996 | monosaccharide metabolic process | 0.00102932686679064 |
| BP | GO:0070936 | protein K48-linked ubiquitination | 0.00121029958826664 |
| BP | GO:0046686 | response to cadmium ion | 0.00132367291074696 |
| BP | GO:1901606 | alpha-amino acid catabolic process | 0.00215476076638869 |
| BP | GO:0009063 | cellular amino acid catabolic process | 0.00341651620081103 |
| BP | GO:0046916 | cellular transition metal ion homeostasis | 0.00372703572411213 |
| BP | GO:0055076 | transition metal ion homeostasis | 0.0053179709575335 |
| BP | GO:0033131 | regulation of glucokinase activity | 0.00798462745638295 |
| BP | GO:0022900 | electron transport chain | 0.00842004831642635 |
| BP | GO:0001967 | suckling behavior | 0.0087798089124913 |
| BP | GO:0045628 | regulation of T-helper 2 cell differentiation | 0.0087798089124913 |
| BP | GO:1903299 | regulation of hexokinase activity | 0.0087798089124913 |
| BP | GO:0002674 | negative regulation of acute inflammatory response | 0.0095743954273646 |
| BP | GO:1901605 | alpha-amino acid metabolic process | 0.0103646739420697 |
| BP | GO:0002087 | regulation of respiratory gaseous exchange by nervous system process | 0.0103683874143559 |
| BP | GO:0035745 | T-helper 2 cell cytokine production | 0.0103683874143559 |
| BP | GO:2000551 | regulation of T-helper 2 cell cytokine production | 0.0103683874143559 |
| BP | GO:0071248 | cellular response to metal ion | 0.0105691702121869 |
| BP | GO:0043650 | dicarboxylic acid biosynthetic process | 0.0111617852865515 |
| BP | GO:1902916 | positive regulation of protein polyubiquitination | 0.0111617852865515 |
| BP | GO:0010273 | detoxification of copper ion | 0.0119545894567744 |
| BP | GO:0035435 | phosphate ion transmembrane transport | 0.0119545894567744 |
| BP | GO:0045064 | T-helper 2 cell differentiation | 0.0119545894567744 |
| BP | GO:1990169 | stress response to copper ion | 0.0119545894567744 |
| BP | GO:0002830 | positive regulation of type 2 immune response | 0.012746800337582 |
| BP | GO:0044065 | regulation of respiratory system process | 0.012746800337582 |
| BP | GO:0009084 | glutamine family amino acid biosynthetic process | 0.0135384183412671 |
| BP | GO:0071241 | cellular response to inorganic substance | 0.0137341294233642 |
| BP | GO:0009068 | aspartate family amino acid catabolic process | 0.0143294438798581 |
| BP | GO:0035743 | CD4-positive, alpha-beta T cell cytokine production | 0.0143294438798581 |
| BP | GO:0061687 | detoxification of inorganic compound | 0.0143294438798581 |
| BP | GO:0000209 | protein polyubiquitination | 0.0149103539913266 |
| BP | GO:0046395 | carboxylic acid catabolic process | 0.0149103539913266 |
| BP | GO:0002523 | leukocyte migration involved in inflammatory response | 0.0151198773651188 |
| BP | GO:0097501 | stress response to metal ion | 0.0151198773651188 |
| BP | GO:0016054 | organic acid catabolic process | 0.0153927605587385 |
| BP | GO:0043543 | protein acylation | 0.0157589949587458 |
| BP | GO:0043576 | regulation of respiratory gaseous exchange | 0.0166989698213855 |
| BP | GO:0045624 | positive regulation of T-helper cell differentiation | 0.0174876296145986 |
| BP | GO:0006541 | glutamine metabolic process | 0.018275698998898 |
| BP | GO:0002726 | positive regulation of T cell cytokine production | 0.0190631783847279 |
| BP | GO:0006817 | phosphate ion transport | 0.0190631783847279 |
| BP | GO:0032753 | positive regulation of interleukin-4 production | 0.0198500681822692 |
| BP | GO:0006520 | cellular amino acid metabolic process | 0.0211353161300244 |
| BP | GO:1902914 | regulation of protein polyubiquitination | 0.0214220806518969 |
| BP | GO:0009065 | glutamine family amino acid catabolic process | 0.0222072041430302 |
| BP | GO:0071280 | cellular response to copper ion | 0.0222072041430302 |
| BP | GO:0051604 | protein maturation | 0.022548586134654 |
| BP | GO:0070979 | protein K11-linked ubiquitination | 0.0229917396839686 |
| BP | GO:0048240 | sperm capacitation | 0.0237756876835808 |
| BP | GO:0002828 | regulation of type 2 immune response | 0.0245590485504694 |
| BP | GO:0030431 | sleep | 0.025341822692976 |
| BP | GO:0043372 | positive regulation of CD4-positive, alpha-beta T cell differentiation | 0.025341822692976 |
| BP | GO:0046394 | carboxylic acid biosynthetic process | 0.0254906229385607 |
| BP | GO:0016053 | organic acid biosynthetic process | 0.0257931513510683 |
| BP | GO:0006536 | glutamate metabolic process | 0.0261240105191815 |
| BP | GO:0032633 | interleukin-4 production | 0.0261240105191815 |
| BP | GO:0032673 | regulation of interleukin-4 production | 0.0261240105191815 |
| BP | GO:0045454 | cell redox homeostasis | 0.0276866288536957 |
| BP | GO:0042092 | type 2 immune response | 0.0284670601768549 |
| BP | GO:0002369 | T cell cytokine production | 0.0292469068134126 |
| BP | GO:0002724 | regulation of T cell cytokine production | 0.0292469068134126 |
| BP | GO:0003016 | respiratory system process | 0.0292469068134126 |
| BP | GO:0032496 | response to lipopolysaccharide | 0.0300215342705166 |
| BP | GO:0006882 | cellular zinc ion homeostasis | 0.0300261691701392 |
| BP | GO:0009595 | detection of biotic stimulus | 0.0300261691701392 |
| BP | GO:0045622 | regulation of T-helper cell differentiation | 0.0308048476535457 |
| BP | GO:2000516 | positive regulation of CD4-positive, alpha-beta T cell activation | 0.0308048476535457 |
| BP | GO:0055069 | zinc ion homeostasis | 0.0315829426698802 |
| BP | GO:0055081 | anion homeostasis | 0.0315829426698802 |
| BP | GO:0071276 | cellular response to cadmium ion | 0.0315829426698802 |
| BP | GO:1901223 | negative regulation of NIK/NF-kappaB signaling | 0.0315829426698802 |
| BP | GO:0010677 | negative regulation of cellular carbohydrate metabolic process | 0.0331373839250252 |
| BP | GO:0032691 | negative regulation of interleukin-1 beta production | 0.0331373839250252 |
| BP | GO:0046688 | response to copper ion | 0.0331373839250252 |
| BP | GO:0002237 | response to molecule of bacterial origin | 0.0333222508163124 |
| BP | GO:0010038 | response to metal ion | 0.0350247324285377 |
| BP | GO:0018205 | peptidyl-lysine modification | 0.0355421285119034 |
| BP | GO:0044282 | small molecule catabolic process | 0.0355421285119034 |
| BP | GO:0002673 | regulation of acute inflammatory response | 0.0377867466497995 |
| BP | GO:0045912 | negative regulation of carbohydrate metabolic process | 0.0377867466497995 |
| BP | GO:0006631 | fatty acid metabolic process | 0.0379966215821009 |
| BP | GO:0009066 | aspartate family amino acid metabolic process | 0.038559608700683 |
| BP | GO:0032692 | negative regulation of interleukin-1 production | 0.038559608700683 |
| BP | GO:0046638 | positive regulation of alpha-beta T cell differentiation | 0.0393318913326578 |
| BP | GO:0006120 | mitochondrial electron transport, NADH to ubiquinone | 0.04010359494911 |
| BP | GO:0043370 | regulation of CD4-positive, alpha-beta T cell differentiation | 0.04010359494911 |
| BP | GO:0030514 | negative regulation of BMP signaling pathway | 0.0408747199531672 |
| BP | GO:0002711 | positive regulation of T cell mediated immunity | 0.0439534418989431 |
| BP | GO:0070534 | protein K63-linked ubiquitination | 0.0439534418989431 |
| BP | GO:0051289 | protein homotetramerization | 0.044721679878882 |
| BP | GO:0006979 | response to oxidative stress | 0.0484463019867184 |
| BP | GO:0032731 | positive regulation of interleukin-1 beta production | 0.0485542348151001 |
| CC | GO:0005759 | mitochondrial matrix | 1.34941727662675e-09 |
| CC | GO:1990204 | oxidoreductase complex | 2.34271952465453e-08 |
| CC | GO:0045239 | tricarboxylic acid cycle enzyme complex | 1.27986689492445e-07 |
| CC | GO:0031304 | intrinsic component of mitochondrial inner membrane | 0.00094246907582198 |
| CC | GO:0031305 | integral component of mitochondrial inner membrane | 0.00094246907582198 |
| CC | GO:0098798 | mitochondrial protein-containing complex | 0.00124988150900474 |
| CC | GO:0000151 | ubiquitin ligase complex | 0.00174395924438826 |
| CC | GO:0032592 | integral component of mitochondrial membrane | 0.00225587056103784 |
| CC | GO:0098573 | intrinsic component of mitochondrial membrane | 0.00230723259080455 |
| CC | GO:0061702 | inflammasome complex | 0.0138279444697585 |
| CC | GO:0009295 | nucleoid | 0.0354220851485034 |
| CC | GO:0042645 | mitochondrial nucleoid | 0.0354220851485034 |
| CC | GO:0031301 | integral component of organelle membrane | 0.0390580926894599 |
| CC | GO:0031300 | intrinsic component of organelle membrane | 0.0447177444130597 |
| MF | GO:0016620 | oxidoreductase activity, acting on the aldehyde or oxo group of donors, NAD or NADP as acceptor | 4.13812115440313e-06 |
| MF | GO:0016903 | oxidoreductase activity, acting on the aldehyde or oxo group of donors | 7.02726734649254e-06 |
| MF | GO:0016747 | acyltransferase activity, transferring groups other than amino-acyl groups | 3.37245667879038e-05 |
| MF | GO:0016746 | acyltransferase activity | 5.36080007429856e-05 |
| MF | GO:0016417 | S-acyltransferase activity | 0.000305085157752741 |
| MF | GO:0061631 | ubiquitin conjugating enzyme activity | 0.000440502878521727 |
| MF | GO:0061650 | ubiquitin-like protein conjugating enzyme activity | 0.000517326430815117 |
| MF | GO:0031625 | ubiquitin protein ligase binding | 0.00200477507136996 |
| MF | GO:0044389 | ubiquitin-like protein ligase binding | 0.00239183738335692 |
| MF | GO:0016407 | acetyltransferase activity | 0.00278443836307513 |
| MF | GO:0016783 | sulfurtransferase activity | 0.00867885358712372 |
| MF | GO:0016668 | oxidoreductase activity, acting on a sulfur group of donors, NAD(P) as acceptor | 0.0112687077771479 |
| MF | GO:0042834 | peptidoglycan binding | 0.0155710403183991 |
| MF | GO:0015295 | solute:proton symporter activity | 0.0207106716817776 |
| MF | GO:0035035 | histone acetyltransferase binding | 0.0215648272232675 |
| MF | GO:1901681 | sulfur compound binding | 0.0217798415469764 |
| MF | GO:1990756 | ubiquitin ligase-substrate adaptor activity | 0.024123105062462 |
| MF | GO:0005504 | fatty acid binding | 0.033449905811276 |
| MF | GO:0051539 | 4 iron, 4 sulfur cluster binding | 0.0359790369196521 |
| MF | GO:0051287 | NAD binding | 0.0476998306565607 |
| MF | GO:0016667 | oxidoreductase activity, acting on a sulfur group of donors | 0.048531897209067 |
