# Supplementary material for: Identification of immune characteristic biomarkers and therapeutic targets in cuproptosis for sepsis by integrated bioinformatics analysis and single-cell RNA sequencing analysis
Source: Heliyon. 2024 Mar 3;10(5):e27379. doi: 10.1016/j.heliyon.2024.e27379 (PMC10943398; doi:10.1016/j.heliyon.2024.e27379)
Supplement: Multimedia component 2 [file mmc2.docx]

**Supplementary files:**

**Supplementary file 2:**

Supplementary Table S2: The KEGG pathway enrichment assessments of DECuGs.

| ID | Description | pvalue | geneID |
| --- | --- | --- | --- |
| hsa00020 | Citrate cycle (TCA cycle) | 1.17165771230668e-07 | DLAT/DLD/DLST/PDHB |
| hsa01200 | Carbon metabolism | 2.92710888855286e-05 | DLAT/DLD/DLST/PDHB |
| hsa00620 | Pyruvate metabolism | 3.05881596776261e-05 | DLAT/DLD/PDHB |
| hsa01100 | Metabolic pathways | 7.49264969435093e-05 | COX11/DBT/DLAT/DLD/DLST/GLS/LIAS/LIPT1/PDHB |
| hsa00010 | Glycolysis / Gluconeogenesis | 0.000163178503440596 | DLAT/DLD/PDHB |
| hsa00640 | Propanoate metabolism | 0.00135664940007453 | DBT/DLD |
| hsa00380 | Tryptophan metabolism | 0.00206675364027123 | DLD/DLST |
| hsa00280 | Valine, leucine and isoleucine degradation | 0.00269265461160701 | DBT/DLD |
| hsa00310 | Lysine degradation | 0.00431611961379764 | DLD/DLST |
| hsa05131 | Shigellosis | 0.00518582934502014 | UBE2D1/UBE2D4 |
| hsa05230 | Central carbon metabolism in cancer | 0.00549228752149115 | GLS/PDHB |
| hsa00471 | D-Glutamine and D-glutamate metabolism | 0.00818841672660375 | GLS |
| hsa04120 | Ubiquitin mediated proteolysis | 0.0201999759288754 | UBE2D1/UBE2D4 |
| hsa04141 | Protein processing in endoplasmic reticulum | 0.029311241747136 | UBE2D1/UBE2D4 |
| hsa00220 | Arginine biosynthesis | 0.0339772838491831 | GLS |
| hsa04964 | Proximal tubule bicarbonate reclamation | 0.0371569994695266 | GLS |
| hsa00630 | Glyoxylate and dicarboxylate metabolism | 0.0482100801598003 | DLD |
